# A Fast Neutron Radiography System Using a High Yield Portable DT Neutron Source

**DOI:** 10.3390/jimaging6120128

**Published:** 2020-11-26

**Authors:** David L. Williams, Craig M. Brown, David Tong, Alexander Sulyman, Charles K. Gary

**Affiliations:** Adelphi Technology Inc., 2003 E. Bayshore Rd, Redwood City, CA 94063, USA; craig@adelphitech.com (C.M.B.); dtong@adelphitech.com (D.T.); info@adelphitech.com (A.S.); cgary@adelphitech.com (C.K.G.)

**Keywords:** fast neutron radiography, FNR, imaging, deuterium-tritium, DT, 14.1 MeV, USAF-1951, modulation transfer function, MTF

## Abstract

Resolution measurements were made using 14.1 MeV neutrons from a high-yield, portable DT neutron generator and a neutron camera based on a scintillation screen viewed by a digital camera. Resolution measurements were made using a custom-built, plastic, USAF-1951 resolution chart, of dimensions 125 × 98 × 25.4 mm^3^, and by calculating the modulation transfer function from the edge-spread function from edges of plastic and steel objects. A portable neutron generator with a yield of 3 × 10^9^ n/s (DT) and a spot size of 1.5 mm was used to irradiate the object with neutrons for 10 min. The neutron camera, based on a ^6^LiF/ZnS:Cu-doped polypropylene scintillation screen and digital camera was placed at a distance of 140 cm, and produced an image with a spatial resolution of 0.35 cycles per millimeter.

## 1. Introduction

Kallmann and Kuhn [1,2] are credited with taking the first neutron radiographs shortly after the discovery of the neutron by Chadwick in 1932. They used a low-yield neutron generator with a yield of 4 × 10^7^ n/s and exposure times of 4 to 5 h [3]. Higher-quality neutron radiography images were then produced by Thewlis [4] using reactors and also by Peters [5] using a higher-yield neutron generator than that used by Kallmann, which yielded images of reasonable quality in 1–3 min [3]. High-yield portable neutron generators offer the promise of being able to take a useful neutron radiography system to the object to be imaged rather than vice versa, potentially enabling use of this technique for non-destructive inspection applications. This early neutron imaging work focused on thermal neutrons, which were either produced by a reactor, or were produced by moderating the neutrons from a neutron generator. Fast neutron radiography (FNR), directly using the 14.1 MeV neutrons arising from the deuterium–tritium (DT) reaction, has been reported using lower-yield portable neutron generators by Andersson [6], and high-yield neutron generators that are not portable, by Wu [7,8]. With incremental improvement in DT set up reported by Bishnoi [9] and Kam [10]. It should be noted that FNR using fast, 2.45 MeV, neutrons arising from the deuterium–tritium (DD) reaction has previously been reported using neutron generators [11,12,13]. However, the DD fusion reaction rate is approximately 1% that of the DT reaction rate [14]. Consequently, more power is required to achieve a given neutron yield, so this technology is not suited to truly portable radiography applications. An ideal source for radiography would be a high-yield, small-spot-size source with a small enough footprint to be transportable. To date, some have said that neutron radiography remains constrained by the lack of availability of high-intensity portable neutron sources [15], without which the object to be imaged must be taken to a reactor or fixed neutron generator source, which is impractical for some applications such as building inspection.

The radiographic contrasts obtained by 14.1 MeV FNR are similar to those obtained with high-energy X-ray and gamma-ray radiography and in general are smaller than those achieved with thermal neutron radiography. However, unlike with X-ray and gamma-ray radiography, the broader contrast latitude allows low-atomic-number materials to simultaneously be observed with heavier metals [16].

Imaging performance using a high-yield, small-spot-size, DT generator is presented. A 10 min exposure time resulted in an image with a resolution of 0.35 cycles per mm. A custom-fabricated plastic phantom imaging object was built to investigate the performance of a neutron imaging camera consisting of a scintillation screen viewed with a cooled digital camera charge-coupled device (CCD) array. The resolution was determined using a USAF-1951 resolution target [17] and also by calculating the modulation transfer function (MTF) by analyzing the edges of object images [18]. These calculations were performed for both steel and plastic. The advantage of the neutron-based technique over X-rays is the ability to image “low-Z” (such as hydrogenous) materials, hence the use of the plastic resolution phantom object.

The advent of portable neutron generators with both high yields, >10^9^ n/s, and small neutron emission spot sizes, of 1–2 mm, could potentially enable wider adoption of this technique.

## 2. Materials and Methods

**Figure 1:**
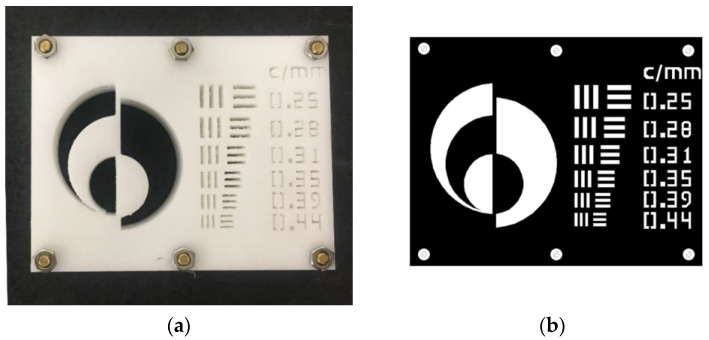
Imaging phantom object—125 mm × 98 mm × 25.4 mm. The spatial frequency of each tri-bar feature is given in cycles per millimeter. (**a**) View from above; (**b**) design.

An imaging phantom object was fabricated from a stack of patterned Delrin^®^ [Polyoxymethylene, (CH_2_O)_n_] sheets with a combined thickness of 25 mm. The sheets were fabricated using a Computer Numerically Controlled (CNC) milling machine patterned with a USAF-1951-compliant resolution chart consisting of different sized vertically and horizontally oriented groups of three bars of equal width and separation. The object also contained six bolt holes which aligned the sheets with respect to each other. The imaging phantom is shown in Figure 1.

**Figure 2:**
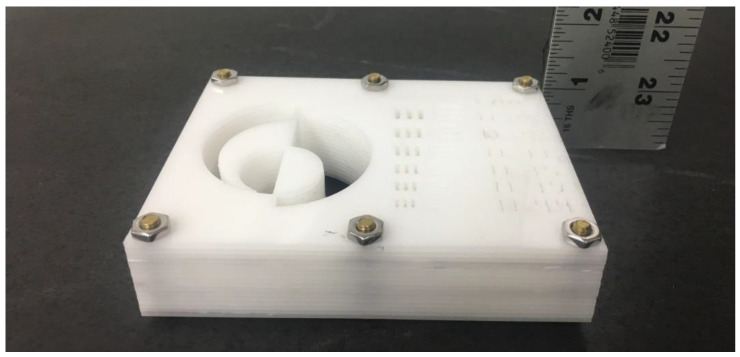
Imaging phantom object—125 mm × 98 mm × 25.4 mm.

The completed object measured 125 mm × 98 mm × 25 mm. The stacked-sheet approach was used because of the fine detail required in the pattern. An object with a feature size less than 1 mm and a depth of 25 mm would otherwise be difficult to fabricate from a single block of plastic since it would require a small-diameter tool (submillimeter diameter) to cut through the entire thickness of the object, likely causing the tool to break. The completed object is shown in Figure 2, which shows the overall thickness of the object.

The imaging phantom object was placed in direct contact with a “250 × 200 mm X-ray Neutron Tomography Camera” from the company Neutron Optics [17], almost flush with the scintillator (aside from the thickness of metal forming the camera’s light-tight box, and a thin sheet of lead used to screen X-rays). The camera consists of a cooled Sony ICX694ALG CCD (2752 × 2200 pixels = 6 Megapixel) that images a 250 mm × 200 mm scintillator using a high-resolution f/1.4 lens. The scintillator is viewed in reflection via a mirror. This configuration allows the camera electronics to be shielded from radiation because it is not in direct line of sight with the neutron source. A 2 mm thick polypropylene phosphor plate, doped with ^6^LiF/ZnS:Cu from RC Tritec Ltd., was used as a fast imaging scintillator. This phosphor emits 530 nm light in response to irradiation with fast (>0.8 MeV) neutrons [19].

The camera was oriented such that the neutron generator’s target was positioned at the scintillator’s zenith at a distance of 140 cm. A 3 mm thickness of lead was placed between the imaging phantom object flush against the camera. This thickness of lead was determined to be effective in screening out unwanted X-rays arising from the neutron generator’s 150 kV acceleration voltage. Lead bricks were placed around the neutron camera’s CCD array to reduce background signals arising from neutron-induced prompt gamma-ray emission from materials in the walls and the floor of the room. The neutron generator and camera were positioned in the center of an empty room, approximately 1 m from the floor. The room had a high roof and a low mass ceiling. Efforts were taken to minimize any mass close to the camera to avoid neutron scatter, and minimize gamma rays produced from neutron interactions with extraneous material in the laboratory. A 20 cm thickness of lithium carbonate was used to attempt to shield the detector from scattered, thermalized neutrons. A compact, portable, Adelphi Technology DT generator was used as a neutron source. The generator was operated with a yield of 3 × 10^9^ n/s DT for 10 min. This yield is estimated from neutron measurements made using a Bonner ball neutron detector. This neutron generator had an emission spot size of 1.5 mm, as observed by measuring the burn pattern on an aluminum foil placed over the solid metal target, prior to loading the generator with tritium. The target was oriented at 45° to both the ion beam and imaging camera, so the projection of the target on the scintillator plane is circular. The expected angular divergence of the beam is consequently L/D = 1400 mm/1.5 mm ≈ 900, otherwise expressed as a divergence angle of Tan^−1^(1.5/1400) = 0.061°.

The USAF resolution chart was analyzed by rotating the image such that the bars were oriented parallel to the axes (i.e., ‘vertical’ lines in the image parallel to the *y* axis and ‘horizontal’ lines parallel to the *x* axis). The average value of the pixels at a given x position was then taken by taking the average value of the pixels in a ‘vertical’ strip. These results were then plotted and the observation of maxima and minima in the expected locations indicates that a given resolution, could be imaged.

Additionally, the modulation transfer function (MTF) was calculated by taking a one-dimensional Fourier transform of the line-spread function (LSF) [20]. The LSF is the derivative of the edge-spread function (ESF), which is the intensity as a function of position along a line perpendicular to the edge of the object. After first rotating the line edge to be parallel to the *y* axis, the x values were averaged across the portion of the image that straddles the edge to derive the LSF. This was smoothed by taking the average of the surrounding pixels over a 2 mm region either side of each point. This was found to be sufficient to allow a useful derivative to be calculated. The resolution of the imaging system is conservatively defined as the reciprocal of the spatial frequency where the modulation transfer function (MTF) falls to 0.15 [21]. The resolution could, alternatively, be reported as the reciprocal of this quantity. These MTF measurements were taken at different locations within the image and compared with the USAF-1951 results.

## 3. Results

**Figure 3:**
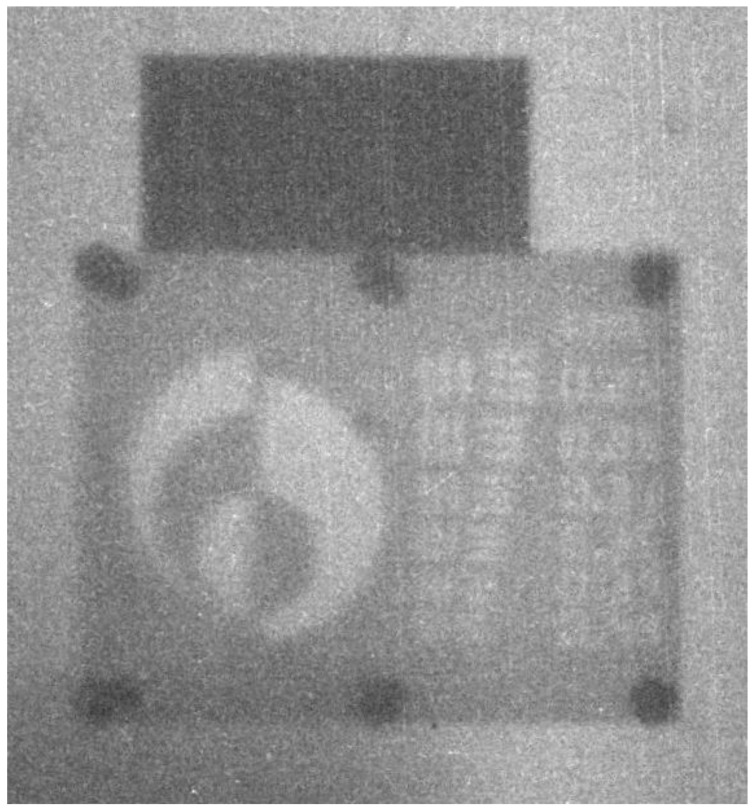
Image from the scintillation camera produced by irradiating the sample for 10 min.

The image shown in Figure 3 was produced when the imaging phantom object was irradiated for 10 min. A 12.5 mm thick steel block was irradiated with the sample for comparison, and is located above the plastic phantom in the image.

Based on the neutron yield, the duration of the exposure and the solid angle subtended by the scintillator from the neutron source, the number of neutrons contributing to the image can be estimated to be (250 × 200 mm)/(4π × 1400^2^ mm^2^) × 3 × 10^9^ n/s × 600 s = 3.7 × 10^9^ neutrons across the 250 × 200 mm imaging area, i.e., 73,000 neutrons per mm^2^.

Variations of overall brightness across the image (darker bottom left corner in Figure 3) were due to the presence of structures within the generator itself. Moving the camera moved the position of the dark spot across the image. Dark-field corrections (background subtraction) was not performed on this image.

### 3.1. Analysis of the USAF-1951 Resolution Chart

#### 3.1.1. Visual Inspection

Inspection of the images shows that a spatial frequency of 0.35 cycles/mm is observable. The results are recorded in Table 1.

**Figure 4:**
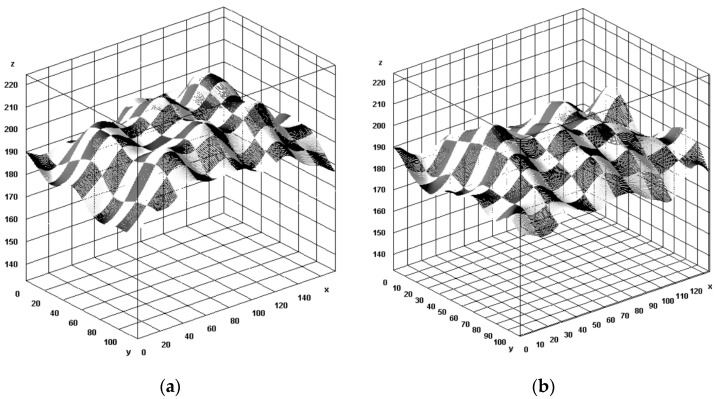
The use of 3D plots can make the images more visible: (**a**) 0.25 c/mm vertical; (**b**) 0.35 c/mm vertical. Units in pixels; averaging was performed to reduce the statistical noise.

Plotting the pixel intensity as a function of position on a 3D graph can make the pattern clearer, as seen in Figure 4.

#### 3.1.2. Brightness as a Function of Position across the USAF-1951 Pattern

**Figure 5:**
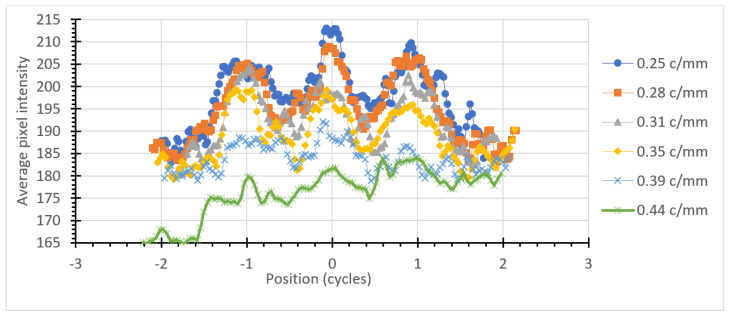
Intensity distribution for the horizontal bars in the USAF-1951 pattern.

**Figure 6:**
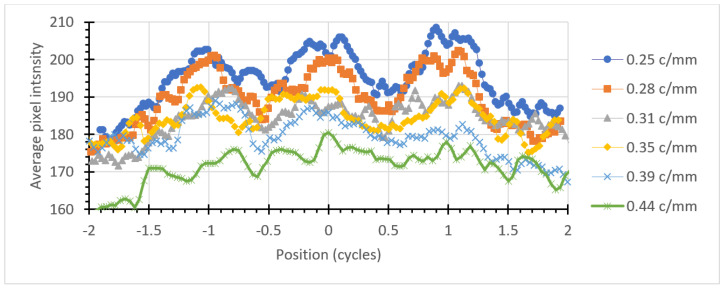
Intensity distribution for the vertical bars in the USAF-1951 pattern.

By averaging the pixel values across each x or y direction, a graph of the average intensity may be constructed. Plotting these on a graph as a function of position in the cycle, rather than absolute distance, allows the three intensity peaks to line up for different line pair spacings in the pattern. This is shown in Figure 5 and Figure 6 for horizontal and vertical bars, respectively.

The graphs shown in Figure 5 and Figure 6 show a central peak and two side lobes corresponding to the three bars comprising each pattern. This periodicity is clearly visible down to 0.35 cycles/mm and is marginal at 0.39 cycles/mm. This is in agreement with visual inspection of the image.

### 3.2. Modulation Transfer Function

**Figure 7:**
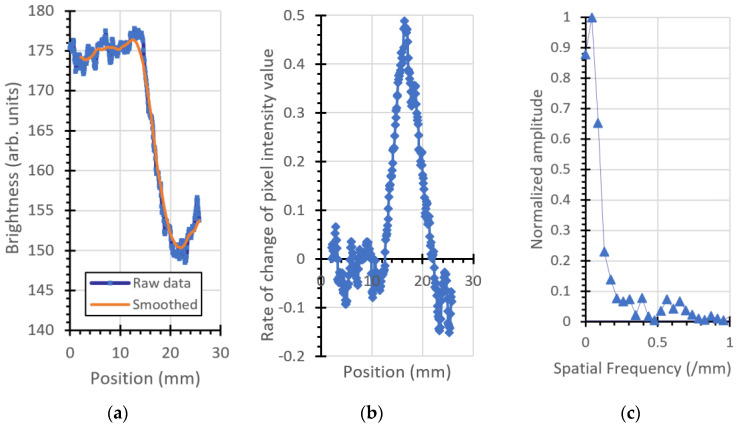
Vertical left edge of the plastic phantom: (**a**) ESF, (**b**) LSF (inverted for clarity), and (**c**) MTF.

**Figure 8:**
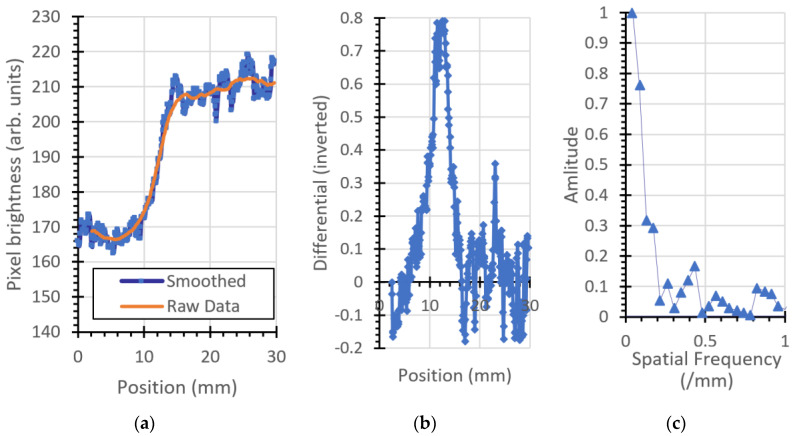
Vertical edge, top part of Adelphi’s logo: (**a**) ESF, (**b**) LSF, and (**c**) MTF.

**Figure 9:**
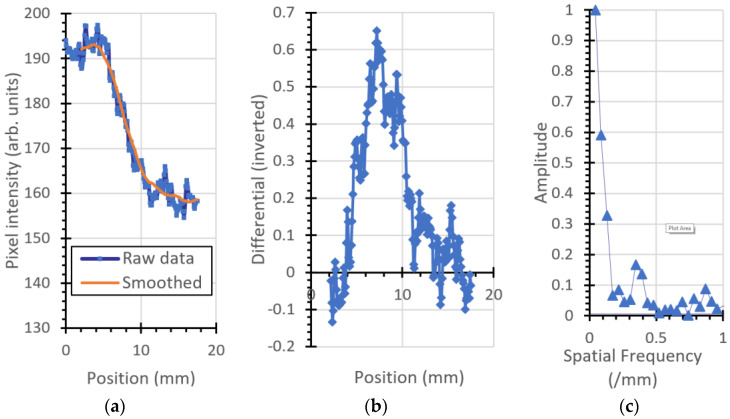
Vertical edge, bottom part of Adelphi’s logo: (**a**) ESF, (**b**) LSF (inverted for clarity), and (**c**) MTF.

**Figure 10:**
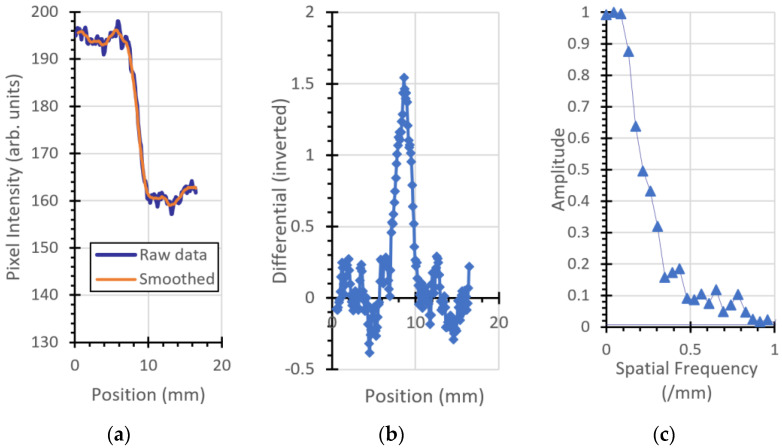
Bottom edge of the phantom: (**a**) ESF, (**b**) LSF (inverted for clarity), and (**c**) MTF.

The ESF, LSF, and MTF for the plastic imaging phantom object are shown in Figure 7, Figure 8, Figure 9 and Figure 10. Figure 7 is the left (vertical) edge of the plastic, Figure 8 and Figure 9 are taken from left to right across the (vertical) transition of Adelphi’s logo and Figure 10 is taken across the bottom (horizontal) edge.

**Figure 11:**
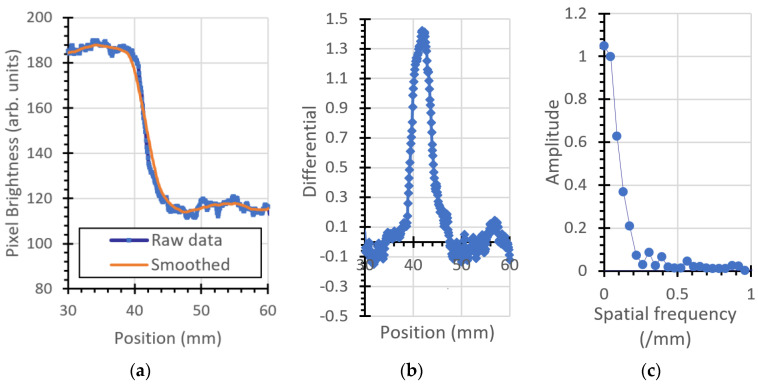
Steel block: (**a**) ESF, (**b**) LSF (inverted for clarity), and (**c**) MTF.

For comparison, the same parameters are plotted for the 12.5 mm thick steel block shown, as shown in Figure 11.

A summary of the results is given in Table 2.

## 4. Discussion

The USAF-1951 image gave a resolution of 0.35 cycles/mm. Measurements of the MTF at various locations within the image provide results of 16 to 18 cycles per mm for vertical lines, and 0.42 cycles per mm for a horizontal line. The best result (0.42 cycles per millimeter) was at the horizontal bottom edge of the phantom (which unfortunately is the only long horizontal edge in the image). The neutron emission spot has a circular projection of diameter 1.5 mm when viewed from the position of the camera (orthogonal to the neutron generator’s ion beam) because the neutron generator’s target is positioned at 45° to the ion beam. The difference between calculated MTF values for horizontal and vertical lines in the object is consequently not attributable to the neutron generator and can be attributed to misalignment of the phantom with respect to the direction of the neutrons, exacerbated by parallax effects. This effect is clearly visible in the optical photograph of the phantom shown in Figure 1a and is particularly noticeable in the curved semicircles forming Adelphi’s logo. The top surface of the phantom is white, the absence of material is black, and the curved semicircular side walls can be seen as a grey color, indicating that the (visible light) camera was not coincident with the projection of the curved surface, as would be the case if the optical camera was positioned at infinity. The same grey color is not observed on the adjacent vertical lines of the logo, as is expected from parallax effects. Consistent with this, the USAF-1951 horizontal tri-bars for 0.31 and 0.39 cycles per millimeter appear darker than the others in the optical image, because of their orientation with respect to the camera. These parallax effects will similarly frustrate the USAF-1951 neutron image and will result in a less pronounced transition in pixel intensity across an interface, resulting in worse MTF measurements.

The origin of the resolution limit in this image is most likely geometrical effects of phantom alignment and parallax caused by the relatively close distance of the detector. The 6 megapixel camera is not the limiting factor. Resolution is likely also hampered by neutron interactions, mostly from the floor and walls of the building.

The experiment is likely under-reporting the achievable resolution. Additional work could lead to improvements:

- Greater number of neutrons in the image, either higher neutron yield (the generator is capable of 1 × 10^10^ n/s) or increase the exposure duration (longer than 10 min);
- Careful alignment of the phantom (taking a series of measurements at slightly different angles to optimize for each structure being imaged);
- Use of a different standard that minimizes the geometrical/parallax effects (for example a spoke wheel);
- Acquiring multiple shorter exposures and adding or averaging them with appropriate filtering methods (e.g., median);
- Improving neutron and gamma-ray shielding around the neutron camera’s scintillator to reduce the effects of scattered neutrons and gamma rays produced by neutron interactions within the scene which subsequently scatter onto the detector;
- Use of a scintillator that is more sensitive to fast neutrons and less sensitive to gamma rays could also be explored.

This work could be extended by repeating this study with imaging phantoms made from different materials in order to illustrate the improved contrast latitude afforded by this technique.

## 5. Conclusions

The resolution of a portable neutron imaging system which was comprised of an Adelphi Technology DT generator and a Neutron Optics camera was investigated. A spatial resolution of 0.35 cycles per mm was measured via a plastic USAF-1951 target. and via MTF calculations, which gave a resolution between 0.16 and 0.42 cycles per mm depending upon the location and orientation of the measurement within the image.

## Figures and Tables

**Table 1:** Visibility of the resolution standard’s tri-bar patterns.

Spatial Frequency ^1^	Horizontal	Vertical	Image
0.25 c/mm	Visible	Visible	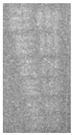
0.28 c/mm	Visible	Visible	
0.31 c/mm	Visible	Visible	
0.35 c/mm	Visible	Visible	
0.39 c/mm	Not visible	Visible	
0.44 c/mm	Not Visible	Not visible	

^1^ Cycles per millimeter.

**Table 2:** MTF measurements from different locations from within the image.

Material	Location	Orientation	Spatial Frequency ^1^
Plastic	Left edge	Vertical	0.17/mm
Plastic	Logo top center	Vertical	0.18/mm
Plastic	Logo bottom, center	Vertical	0.16/mm
Plastic	Bottom edge	Horizontal	0.42/mm
Steel	Left edge	Vertical	0.2/mm

^1^ Smallest spatial frequency at which the MTF is 0.15.
